# Difficulty in Diagnosing Cesarean Scar Pregnancy Using Magnetic Resonance Imaging: Importance of Color Doppler Assessment in Treatment Decision-Making

**DOI:** 10.7759/cureus.117077

**Published:** 2026-09-28

**Authors:** Kaoru Yamada, Keita Hasegawa, Kaori Suenaga, Yoshiyuki Mochimaru, Toru Arase

**Affiliations:** 1 Department of Obstetrics and Gynecology, Keiyu Hospital, Yokohama, JPN

**Keywords:** cesarean scar pregnancy, color doppler ultrasonography, ectopic pregnancy (ep), magnetic resonance imaging (mri), uterine artery embolization (uae)

## Abstract

Cesarean scar pregnancy (CSP) is a rare but potentially life-threatening form of ectopic pregnancy, and its diagnosis may be challenging in early pregnancy. We report a case of a 40-year-old woman with a history of one cesarean section who was referred with suspected cervical pregnancy. Transvaginal ultrasonography demonstrated a cystic lesion at the cesarean scar site without detectable blood flow, whereas magnetic resonance imaging (MRI) suggested right adnexal ectopic pregnancy. Laparoscopic surgery was initiated; however, no adnexal pregnancy was identified, and the procedure was converted to ultrasound-guided uterine evacuation without preoperative uterine artery embolization. Chorionic villi were confirmed pathologically, and the postoperative course was uneventful. This case highlights that MRI findings may be atypical in early CSP and that color Doppler ultrasonography of peritrophoblastic blood flow may be useful for treatment planning, including decisions on preoperative uterine artery embolization.

## Introduction

Cesarean scar pregnancy (CSP) is a form of ectopic pregnancy in which the gestational sac implants within the uterine scar from a previous cesarean section. The estimated prevalence is ~1 in 1,500-2,000 pregnancies among women with a previous cesarean delivery, and its incidence is increasing in parallel with rising cesarean delivery rates worldwide [1,2]. As CSP may lead to serious maternal morbidity, including severe vaginal or intra-abdominal bleeding, uterine rupture, and the development of placenta accreta spectrum disorders, careful evaluation is essential.

The diagnosis of CSP relies primarily on transvaginal ultrasonography; however, imaging findings may be atypical in very early pregnancy, making it difficult to distinguish CSP from cervical pregnancy, incomplete miscarriage, or adnexal ectopic pregnancy [3]. Magnetic resonance imaging (MRI) can serve as a complementary diagnostic tool; however, its value may be limited in early cases.

Assessment of hemorrhage risk is also important in the management of CSP. Contrast-enhanced MRI enables the evaluation of lesion enhancement patterns. Additionally, the assessment of lower uterine segment thickness and gestational mass diameter using transvaginal ultrasonography may be useful for predicting intraoperative hemorrhage risk and determining the need for preoperative uterine artery embolization (UAE) [4]. Furthermore, CSP is commonly classified as on-the-scar (type 1) or in-the-niche (type 2), which may determine clinical outcomes and treatment selection [5]. However, because there are no universally accepted treatment guidelines, management strategies vary widely, from systemic or local methotrexate administration to surgical approaches, including uterine evacuation, hysteroscopic resection, and laparoscopic or open excision, with or without preoperative UAE [6,7].

Herein, we report a case of CSP in which MRI findings were inconclusive for diagnosis. However, the absence of vascular flow on transvaginal color Doppler ultrasonography allowed the omission of preoperative UAE, resulting in minimal intraoperative bleeding, and the lesion was successfully managed using a suction technique.

## Case presentation

A 40-year-old Japanese woman (gravida 3, para 1) with a history of one previous cesarean section and one spontaneous abortion was referred to our hospital at six weeks of gestation for suspected cervical pregnancy. The pregnancy had been achieved following timed intercourse. Although her serum human chorionic gonadotropin (hCG) level was 1,237 mIU/mL at five weeks and two days of gestation, no gestational sac was visible. At six weeks and one day, the hCG level had risen to 3,887 mIU/mL; however, an intrauterine gestational sac was still not identified. Instead, a hypoechoic lesion suggestive of a gestational sac was detected within the cervical canal, prompting referral to our hospital.

Upon examination, transvaginal ultrasonography revealed an endometrial thickness of 11.5 mm and a cystic lesion without detectable blood flow on color Doppler ultrasonography at the cesarean scar site (Figure 1a). The left ovary appeared normal, and no ascites were present. However, a right adnexal mass with ring-shaped vascular flow was observed. MRI findings were considered consistent with either a right tubal or ovarian pregnancy. Little fluid collection was observed at the cesarean scar site, without early contrast enhancement or hemorrhagic change, which was considered atypical for a gestational sac (Figure 1b).

**Figure 1:**
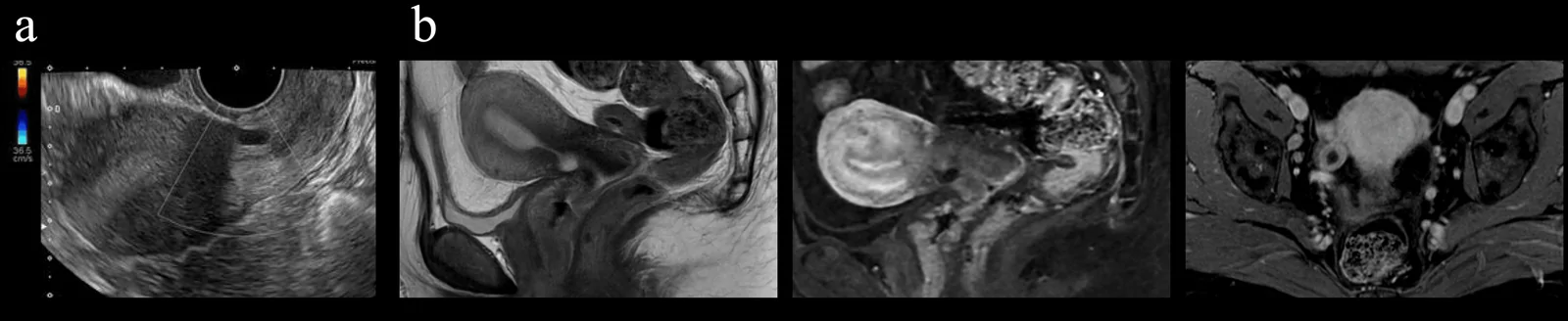
Preoperative imaging (a) Preoperative transvaginal ultrasonography revealing a cystic lesion at the cesarean scar site without detectable blood flow on color Doppler ultrasonography. (b) Preoperative MRI. T2-weighted sagittal image (left) showing a small amount of fluid collecting at the cesarean scar site; contrast-enhanced T1-weighted sagittal image (middle) demonstrating no early contrast enhancement or hemorrhagic change; and contrast-enhanced T1-weighted axial image (right) showing a right adnexal mass.

Based on these findings, the patient underwent laparoscopic surgery with a planned right salpingectomy or partial oophorectomy, combined with uterine evacuation. Methotrexate was not selected because the diagnosis remained uncertain and the right adnexal mass appeared to represent a viable-appearing ectopic pregnancy, for which direct surgical assessment was considered appropriate. Intraoperatively, the right fallopian tube showed no evidence of swelling, and only a corpus luteum was observed in the right ovary (Figure 2a). Consequently, the procedure was converted to uterine evacuation under transabdominal ultrasound guidance. Evacuation was completed without uterine perforation, and villous tissue was macroscopically identified in the evacuated specimen (Figure 2b). Postoperative transvaginal ultrasonography confirmed complete disappearance of the cystic lesion at the cesarean scar site. Final laparoscopic inspection demonstrated no bleeding from the right adnexa.

**Figure 2:**
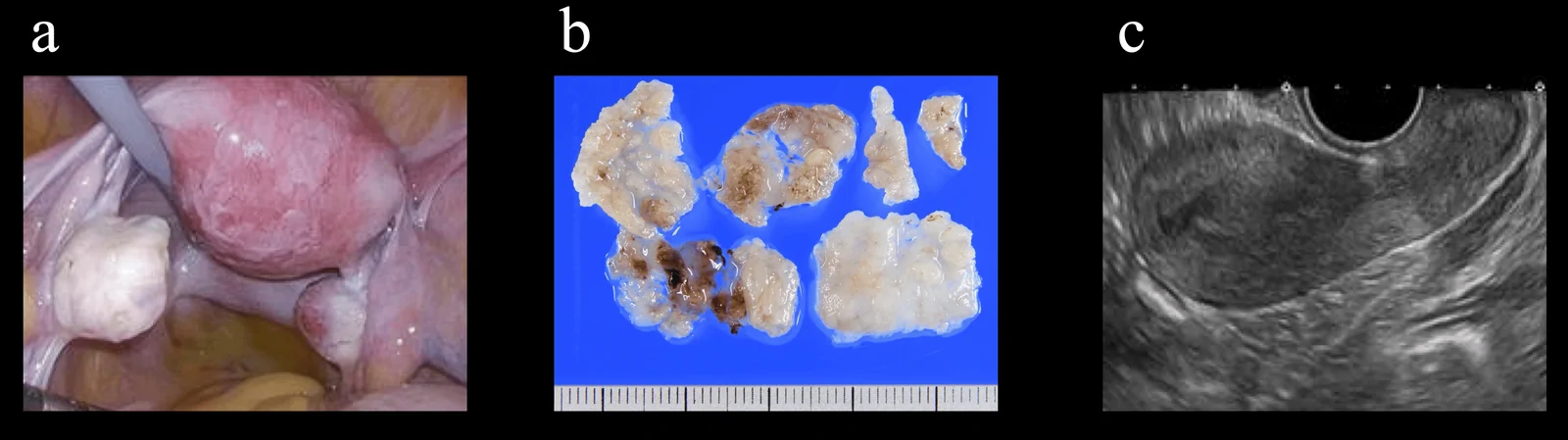
Intraoperative findings, gross appearance of the specimen, and postoperative imaging (a) Intraoperative laparoscopic findings showing no swelling of the right fallopian tube and only a corpus luteum in the right ovary. (b) Macroscopic appearance of the evacuated specimen containing chorionic villi. (c) Postoperative transvaginal ultrasonography at the two-week follow-up showing resolution of the cystic lesion at the cesarean scar site.

The postoperative serum hCG level decreased to 2,373 mIU/mL, and the patient was discharged on postoperative day 4 after an uneventful recovery. At the two-week follow-up, the endometrial thickness decreased to 5.9 mm, both adnexa appeared normal, and the serum hCG level declined to 14.8 mIU/mL (Figure 2c). Pathological examination confirmed chorionic villi without molar or other abnormal pathological findings. At the two-month follow-up, the endometrium and both adnexa appeared normal, and the serum hCG level was negative (Table 1).

**Table 1:** Changes in serum human chorionic gonadotropin (hCG) levels over time

Parameters (units)	Values at 5 weeks 2 days of gestation	Values at 6 weeks 1 day of gestation	Values on postoperative day 1	Values on postoperative day 14	Values at postoperative month 2	Reference value
hCG, mIU/mL	1237	3887	2373	14.8	<5	<5

## Discussion

This case highlights two important clinical points. First, the diagnosis of CSP may be challenging even with MRI. Second, the assessment of peritrophoblastic blood flow using transvaginal color Doppler ultrasonography may be useful for selecting the optimal treatment strategy, including the selection of preoperative UAE.

Transvaginal ultrasonography is considered the first-line imaging modality for the diagnosis of CSP. Timor-Tritsch et al. proposed the following diagnostic criteria: an empty uterine cavity and cervical canal, a gestational sac located in the anterior uterine wall within the cesarean scar, a thin or absent myometrial layer between the gestational sac and bladder, and abundant peritrophoblastic blood flow on color Doppler imaging [1,2]. In the present case, transvaginal ultrasonography at the first visit to our hospital demonstrated a cystic lesion at the presumed cesarean scar site. However, the lesion showed no detectable blood flow on color Doppler imaging. In contrast, a mass-like lesion with ring-shaped blood flow was observed in the right adnexal region. Based on the ultrasonographic findings alone, right adnexal ectopic pregnancy was suspected rather than CSP. MRI demonstrated little fluid collection at the cesarean scar site, with no early contrast enhancement or hemorrhagic change, and the lesion was interpreted as atypical for a gestational sac. Consequently, a right tubal or ovarian pregnancy was suspected. Thus, neither transvaginal ultrasonography nor MRI demonstrated findings typical of CSP, and a definitive preoperative diagnosis could not be established. In early CSP, color Doppler imaging may not always demonstrate abundant blood flow because trophoblastic vascularity may still be immature. Similarly, MRI findings of a gestational sac may be atypical in very early pregnancy. This case illustrates a diagnostic pitfall of CSP and emphasizes that clinicians should maintain a high index of suspicion in early pregnancy, particularly in patients with a history of cesarean delivery, even when imaging findings are atypical [1].

There is no universally established treatment strategy for CSP. Various options have been reported, including uterine evacuation, hysteroscopic resection, laparoscopic or open resection of the scar pregnancy, systemic or local methotrexate administration, and surgery combined with preoperative UAE [6,7]. According to the Society for Maternal-Fetal Medicine guidelines, UAE or temporary uterine artery occlusion may be performed before surgical treatment to reduce the risk of massive bleeding [8]. Color Doppler assessment of peritrophoblastic blood flow may be useful for predicting intraoperative hemorrhage risk; abundant vascularity is considered a risk factor for massive bleeding, whereas poor or absent blood flow may indicate a relatively low hemorrhagic risk [4]. In the present case, preoperative UAE was not performed for two reasons: right adnexal ectopic pregnancy rather than CSP was suspected preoperatively, and no peritrophoblastic blood flow was detected on color Doppler imaging. Therefore, laparoscopic surgery was initiated. Intraoperatively, there was no swelling of the right fallopian tube, and only a corpus luteum was observed in the right ovary. Accordingly, CSP was suspected intraoperatively, and the procedure was converted to uterine evacuation under transabdominal ultrasound guidance. There was no uterine perforation or massive bleeding. Chorionic villi were macroscopically identified in the evacuated tissue, and the postoperative decline in serum hCG confirmed successful treatment. In retrospect, the absence of peritrophoblastic blood flow on color Doppler imaging, together with the relatively low serum hCG level and early gestational age, was consistent with a low risk of intraoperative hemorrhage. This case suggests that color Doppler assessment is important in the diagnosis of CSP and treatment decision-making, particularly when determining the necessity of preoperative UAE [4].

Another notable feature of this case was the discrepancy between the preoperative and final diagnoses. The patient had a history of cesarean section and miscarriage, both of which are risk factors for CSP. The referring physician initially suspected cervical pregnancy because the cystic lesion was located near the cervical canal. However, this finding can also be observed in CSP. CSP may be mistaken for cervical pregnancy, miscarriage in progress, or a low-lying intrauterine pregnancy. Rotas et al. reported that ~13.5% of CSP cases were initially misdiagnosed [3]. Therefore, when a cystic lesion is identified in the lower uterine segment or near the cervical canal during early pregnancy in a patient with a history of cesarean section, CSP should be strongly considered in the differential diagnosis. Evaluation using multiple imaging modalities and early referral to a specialized center are important. In the present case, a hybrid approach combining laparoscopy and transabdominal ultrasonography was useful. Laparoscopy allowed the exclusion of adnexal pregnancy while providing real-time monitoring of the uterine serosal surface. Additionally, transabdominal ultrasound guidance enabled careful removal of the gestational tissue and helped avoid uterine perforation and massive bleeding. This case suggests that CSP can mimic cervical pregnancy in early pregnancy. When the diagnosis is uncertain, laparoscopic assessment combined with ultrasound-guided uterine evacuation may be useful for diagnosis and treatment.

This report is limited by its single-case design; therefore, the findings cannot be generalized to all patients with CSP. In addition, quantitative Doppler parameters, such as peak systolic velocity and resistance index, were not measured. Further studies involving larger numbers of patients are needed to validate these findings. 

## Conclusions

Early CSP may have inconclusive MRI and color Doppler findings. In patients with a previous cesarean delivery, clinicians should maintain diagnostic suspicion when a lesion is identified near the scar. Careful Doppler assessment may help estimate hemorrhage risk and inform individualized treatment planning.
